# Profiling of Urinary Glucuronidated Bile Acids across Age Groups

**DOI:** 10.3390/metabo12121230

**Published:** 2022-12-07

**Authors:** Yamato Muto, Mitsuyoshi Suzuki, Genta Kakiyama, Takahiro Sasaki, Tsuyoshi Murai, Hajime Takei, Hiroshi Nittono

**Affiliations:** 1 Department of Pediatrics, Faculty of Medicine, Juntendo University, Tokyo 113-8421, Japan; 2 Department of Internal Medicine, School of Medicine, Virginia Commonwealth University, 1101 E. Marshall St. Sanger Hall Suite 1-030, Richmond, VA 23298, USA; 3 Hunter Holmes McGuire Veterans Affairs Medical Center, 1201 Broad Rock Blvd., Richmond, VA 23249, USA; 4 Faculty of Pharmaceutical Sciences, Health Sciences University of Hokkaido, 1757 Kanazawa, Tohbetsu-cho, Ishikari, Hokkaido 061-0293, Japan; 5 Junshin Clinic Bile Acid Institute, 2-1-22 Haramachi, Meguro-ku, Tokyo 152-0011, Japan

**Keywords:** glucuronidation, hydrophilic bile acids, uridine 5′-dipospho-glucuronosyltransferase, detoxification, urinary expression

## Abstract

We investigated the age-dependent changes in urinary excretion of glucuronidated bile acids at the C-3 position. Bile acid 3-glucuronides accounted for 0.5% of urinary bile acids in neonates, and the proportion of bile acid 3-glucuronides plateaued at 1–3 years of age. The 3-glucuronides of secondary bile acids were first secreted at 3 months of age, the same time as the establishment of the gut bacterial flora in infants. A considerable portion of bile acid 3-glucuronides were present as non-amidated forms. Our results indicate dynamic hepatic enzyme activity in which the levels of uridine 5′-diphospho-glucuronosyltransferases (UGTs) differ by age group, with higher glucuronidation activity of UGTs towards nonamidated bile acids than amidated bile acids.

## 1. Introduction

Glucuronidation, catalyzed by uridine 5′-diphospho-glucuronosyltransferase (UGT) enzymes, is often involved in drug metabolism and the detoxification of cholestatic bile acids. This conjugation reaction transfers a highly hydrophilic glucuronide group to hydrophobic molecules, resulting in metabolites that become less toxic and are more easily excreted in urine as glucuronide products. To date, the ontology of some hepatic UGT isoforms such as UGT1A and UGT2B has been evaluated based on their glucuronidation activity, mRNA levels, and/or protein abundance in human liver [1,2]. Interestingly, hepatic glucuronidation activity has been shown to be lower in children than in adults [1], and serum levels of bile acid glucuronides differ by sex and genetic makeup [1,3]. However, the age-dependent changes of both serum and urinary glucuronidated bile acid levels have remained unclear.

Recently, we reported the physiologic patterns of urinary bile acid excretion across age groups [4]. However, we did not characterize the patterns of glucuronidated bile acids due to the unavailability of authentic standards at that time. In humans, bile acids predominantly form 3α-*O*-glucuronide, except some minor bile acids such as hyodeoxycholic acid (HDCA) that forms 6α-*O*-glucuronide [5]. We have synthesized authentic standards for 17 kinds of bile acid 3-glucuronides (Supplementary Table S1) whose urinary concentrations across age groups have not been previously traced. The aim of this study was to understand the age-dependent changes of urinary glucuronidated bile acid levels and to clarify the reference ranges using healthy samples.

## 2. Methods

The chemical structure and purity (≥95%), of the synthesized bile acid 3-glucuronides were confirmed by nuclear magnetic resonance [6]. Of the 92 healthy urine samples analyzed in our previous report [4], 69 samples, ranging from those of neonates to those of adults (0 to 58 years of age, 35 males), were available for this additional analysis. These 69 specimens had been aliquoted in separate tubes and kept frozen at −20 °C since the time of their collection. Urine samples were collected at Junshin Clinic or Juntendo University Hospital from January 2017 to December 2019. 

We quantified the 17 kinds of bile acid 3-glucuronides, as well as 66 other types of bile acids, including sulfated, unconjugated, and polyhydroxylated bile acids, and metabolic intermediary bile acids, with the same experimental procedure [4] (Supplemental Table S1). The significance of sex differences in total bile acids, composition, and conjugation rates was calculated for every age group (unpaired *t*-test). Values of *p* < 0.05 were accepted as significant. This study was approved by the Juntendo University Institutional Review Board (approval numbers 16–191, 24–513), and informed consent was obtained from the patients’ parents prior to study enrollment. The study was also in compliance with the 1964 Declaration of Helsinki and its later amendments (as revised in Edinburgh 2000) or comparable ethical standards.

## 3. Results

### 3.1. Age-Dependent Changes in Urinary Excretion Forms of Bile Acids

Table 1 presents the concentrations and composition of urinary bile acids by age group. No sex differences were identified in total bile acids, composition, or conjugation rate in any age group. As in our previous report [4], the total bile acid concentration was higher in the neonatal period (0 to 2 months of age) due to the presence of physiological cholestasis, and it decreased gradually with age. Throughout all age groups, glucuronidated bile acids accounted for only a minor proportion of the total urinary bile acids. The total glucuronidated bile acids (sum of the amidated and nonamidated bile acid-3-glucuronides) accounted for only 0.5% of the urinary bile acids in the neonatal period. This was much smaller than the proportion of sulfated bile acids in the same age group, which accounted for approximately 10% of the urinary bile acids. Subsequently, the percentage of bile acid glucuronides increased gradually and reached a plateau at 1 to 3 years of age (approximately 10% of the total bile acids). Meanwhile, the sulfated bile acids consistently accounted for more than 50% of the urinary bile acids after 3 months of age.

### 3.2. Changes in Urinary Glucuronidated Bile Acids across Age Groups

**Figure 1:**
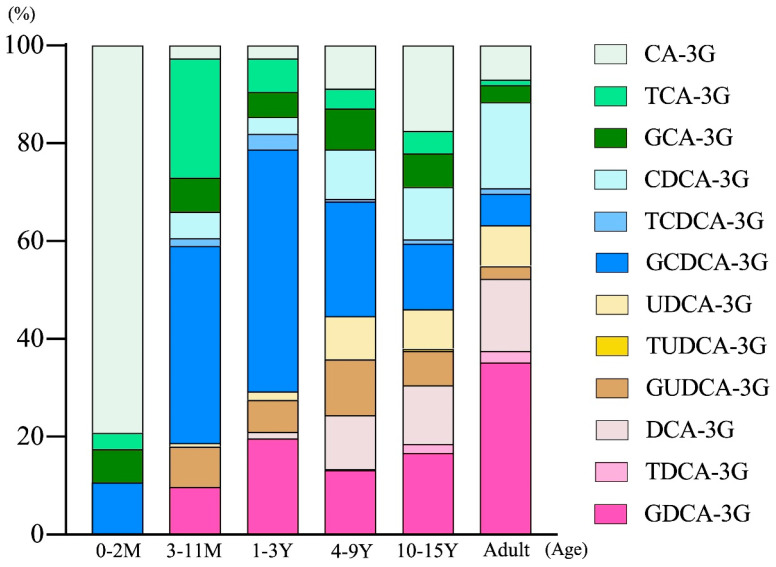
Changes in urinary glucuronidated bile acids across age groups.

Figure 1 presents the differences in the composition of bile acid 3-glucuronides across age groups. In the neonatal period (0 to 2 months of age), 80% of the bile acid 3-glucuronides consisted of nonamidated cholic acid (CA)-3-glucuronide, and the remaining 20% consisted of 3-glucuronides of taurocholic acid (TCA), glycocholic acid (GCA), and glycochenodeoxycholic acid (GCDCA). This high proportion of nonamidated CA-3-glucuronide decreased rapidly in the period of 3–11 months of age. Instead, the proportion of GCDCA-3-glucuronide increased dramatically in this period. In the infantile period (1–3 years of age), GCDCA-3-glucuronide accounted for 40% to 50% of the total bile acid glucuronides. With the increase in secondary bile acid generation in the gut, the proportion of primary bile acid glucuronides decreased gradually. In adult specimens, the 3-glucuronides of CA and CDCA with their taurine/glycine conjugates accounted for approximately 40% of the total bile acid 3-glucuronides. Meanwhile, the proportion of secondary bile acid glucuronides, including deoxycholic acid (DCA)-3-glucuronide, and ursodeoxycholic acid (UDCA)-3-glucuronide with their taurine/glycine conjugates, started increasing at 3–11 months of age. This timing coincided with the elevation of other conjugated secondary bile acids, such as GDCA-3-sulfate (4). Of note, neither amidated nor nonamidated types of lithocholic acid (LCA)-3-glucuronide were found in any age group. Subsequently, the proportion of glucuronides of secondary bile acids continued to increase. In adult specimens, 3-glucuronides of DCA and UDCA and their taurine/glycine conjugates accounted for approximately 60% of the total bile acid 3-glucuronides. It is noteworthy that a considerable portion of bile acid 3-glucuronides was found in non-amidated forms across all age groups (Table 1). In contrast, urinary bile acid 3-sulfates were predominantly found in amidated forms. This observation suggests a difference in the substrate specificity between UGTs and cytosolic steroid sulfotransferases.

Primary bile acids, CA-3G and CDCA-3G, were detected at 0–2 months, with CDCA-3G levels increasing after 3 months of age to become the predominant bile acid. Secondary bile acids, UDCA-3G and DCA-3G, were also observed after 3 months of age. GLCA-3G, TLCA-3G, LCA-3G, HCA-3G, and HDCA-3G were not detected in this study. Abbreviations are defined in Supplemental Table S1.

## 4. Discussion

It has been reported that the expressions of *UGT1A* and *UGT2B* can be detected in fetal liver at as early as 20 weeks of gestational age [2]. Normally, the protein expression levels of UGT1As and UGT2Bs remain low in the neonatal liver, and they gradually increase with age [2]. A study using an allosteric ontogeny equation showed that the ages at which 50% abundance (Age_50_) is reached for UGT1A1, UGT1A4, UGT1A6, UGT1A9, and UGT2B7 are 7.5, 3.6, 10.3, 8.2, and 2.8 years, respectively [1]. In the present data, the proportion of bile acid 3-glucuronides plateaued at 1 to 3 years of age (approximately 10% of total bile acids). Since glucuronidation at the C-3 position of bile acid molecules appears to be catalyzed by hepatic UGT1A4 and UGT2B7 [5], the trends in the urinary secretion of bile acid 3-glucuronides corresponded to the age-dependent activities of hepatic enzymes. It is important that more sulfated bile acids than glucuronidated bile acids were present across all age groups, which accounted for more than 50% of the total urinary bile acids (after 3 months of age). In general, urinary bile acid concentration is up to 10-fold higher with more hydrophilic properties than in plasma [7]. Since adding a sulfoxy group makes the bile acid molecule more hydrophilic than adding glucuronic acid to it, the profile of dominant sulfated bile acid likely represents a physiological way of the body to excrete bile acids into the urine.

It should be mentioned that the present study analyzed only the C3 position of glucuronidated bile acids. In human urine, the presence of acyl glucuronidated bile acids, which are conjugated through the terminal carboxy group of bile acids at C-24, has also been reported [8,9]. In serum, CDCA-3-glucuronide and DCA-3-glucuronide, in addition to the less abundant hyocholic acid (HCA)-6-glucuronide and HDCA-6-glucuronide, are found as the major bile acid glucuronides [5]. The 3-glucuronides and 6-glucuronide bile acids represent more than 95% of the total serum bile acid glucuronides [5]. Therefore, the amounts of urinary acyl glucuronides of bile acids may be negligible when compared to the amounts of 3-glucuronides and 6-glucuronides of bile acids. It should also be mentioned that the human kidney has a significant drug glucuronidation capability [9,10]. The rate of bile acid glucuronidation in the kidney, the substrate specificity of kidney UGTs, and the predilection for glucuronidation (C-3, C-24, or other) by kidney UGTs are largely unknown. Further investigations of these points are clearly necessary.

Limitations of the study included the (1) small sample size and high variability in bile acid levels when comparing across age groups, (2) the lack of a controlled diet by the patients (e.g., low fat versus high fat) before the sample collection, and (3) the lack of assessment of the impact of the circadian rhythm on bile acid levels. Although detailed correlative investigations between organ-specific UGT activities and the urinary excretion pattern of each bile acid glucuronide are necessary, the data on urinary bile acid 3-glucuronides in this study appear to reflect the activity of age-dependent UGTs. The urinary profile of these glucuronidated bile acids, in addition to the other conjugated bile acids in our previous report [4], covers all major types of bile acids and their physiological patterns of urinary excretion across all age groups. These data will serve as a reference for age-dependent hepatobiliary diseases and/or high-risk screening for inborn errors of bile acid metabolism. For instance, Rotor syndrome, which is characterized by deficiency of organic anion transporting polypeptide (OATP) 1B1 and OATP1B3, can be diagnosed by examining the ratio of the bile acid 3-glucuronides. In these patients, the 3-glucuronides are unable to enter hepatocytes through OATP1B1/B3 and subsequently accumulate in the circulating blood and are preferentially excreted through the urine, accounting for 20–30% of total BA in the urine and 50–70% in the serum [11].

In summary, we showed for the first time the changes in urinary bile acid 3-glucuronide levels across different age groups. The major observations were: (1) bile acid 3-glucuronide levels increased gradually after birth until the age of 1 year, and accounted for less than 10% of the urinary bile acids throughout later life; (2) the 3-glucuronides of secondary bile acids are secreted into urine at as early as 3 months of age, the same time as the establishment of the gut bacterial flora in infants; and (3) considerable portions of bile acid 3-glucuronides were found in nonamidated forms across all age groups, suggesting a higher glucuronidation activity of UGTs towards nonamidated bile acids than amidated bile acids.

## 5. Conclusions

In this study, urinary secretion of bile acid 3-glucuronides was found to be minimal (0.5% of urinary bile acids) in neonates and increased to 10% by 1–3 years of age, reflecting hepatic UGT activity with age. Furthermore, a considerable portion of urinary bile acid 3-glucuronides was demonstrated to be present as nonamidated forms regardless of age, suggesting higher glucuronidation activity of UGTs towards nonamidated bile acids than amidated bile acids.

## Figures and Tables

**Table 1:** Age-dependent changes in the forms of bile acids excreted in the urine *^1^.

	**0–2 m**		**3–11 m**		**1–3 y**	
	**(*n* = 10, male: 7)**		**(*n* = 22, male: 9)**		**(*n* = 12, male: 6)**	
	Mean ± SD *^4^	**%**	**Mean ± SD**	**%**	**Mean ± SD**	**%**
3-Glucuronidated						
24-Nonamidated	0.04 ± 0.11	0.38	0.01 ± 0.01	0.23	0.01 ± 0.01	1.09
24-Glycine	0.01 ± 0.02	0.08	0.09 ± 0.12	3.02	0.11 ± 0.21	8.59
24-Taurine	0.00 ± 0.01	0.02	0.03 ± 0.04	0.90	0.01 ± 0.01	0.64
3-Sulfated						
24-Nonamidated	0.04 ± 0.06	0.35	0.02 ± 0.02	0.75	0.01 ± 0.03	1.09
24-Glycine	0.65 ± 0.36	5.97	0.79 ± 0.34	25.96	0.51 ± 0.53	39.06
24-Taurine	0.47 ± 0.46	4.36	0.24 ± 0.17	7.79	0.08 ± 0.07	5.95
3-Unconjugated						
24-Nonamidated	012 ± 0.16	1.14	0.12 ± 0.22	3.93	0.03 ± 0.03	2.03
24-Glycine	0.78 ± 1.09	7.18	0.27 ± 0.15	8.73	0.21 ± 0.39	15.76
24-Taurine	0.39 ± 0.57	3.58	0.13 ± 0.16	4.16	0.01 ± 0.02	1.08
Polyhydroxylated *^2^	6.74 ± 0.01	61.85	0.53 ± 0.42	17.23	0.07 ± 0.11	5.33
Others *^3^	1.64 ± 0.94	15.09	0.83 ± 0.44	27.30	0.25 ± 0.17	19.40
**Total**	10.89 ± 14.23	100	3.05 ± 1.28	100	1.31 ± 1.59	100
	**4–9 y**		**10–15 y**		**Adult**	
	**(*n* = 13, male: 7)**		**(*n* = 7, male: 4)**		**(*n* = 5, male: 2)**	
	**Mean ± SD**	**%**	**Mean ± SD**	**%**	**Mean ± SD**	**%**
3-Glucuronidated						
24-Nonamidated	0.02 ± 0.02	2.55	0.03 ± 0.05	5.97	0.02 ± 0.01	2.81
24-Glycine	0.04 ± 0.01	4.85	0.03 ± 0.03	5.62	0.04 ± 0.05	6.48
24-Taurine	0.00 ± 0.00	0.36	0.00 ± 0.01	0.71	0.00 ± 0.00	0.14
3-Sulfated						
24-Nonamidated	0.34 ± 0.34	1.13	0.02 ± 0.02	2.93	0.01 ± 0.01	1.49
24-Glycine	0.05 ± 0.06	46.41	0.26 ± 0.36	46.54	0.39 ± 0.44	62.05
24-Taurine	0.02 ± 0.02	6.95	0.05 ± 0.08	8.99	0.03 ± 0.02	4.23
3-Unconjugated						
24-Nonamidated	0.05 ± 0.03	2.99	0.02 ± 0.03	4.11	0.02 ± 0.01	2.60
24-Glycine	0.01 ± 0.00	7.40	0.02 ± 0.02	3.89	0.01 ± 0.02	1.99
24-Taurine	0.01 ± 0.00	0.82	0.00 ± 0.00	0.51	0.00 ± 0.00	0.00
Polyhydroxylated *^2^	0.02 ± 0.03	3.16	0.01 ± 0.01	1.30	0.00 ± 0.00	0.34
Others *^3^	0.17 ± 0.09	23.38	0.11 ± 0.05	19.42	0.11 ± 0.04	17.87
**Total**	0.74 ± 0.61	100	0.56 ± 0.68	100	0.63 ± 0.60	100

Data are shown as means ± standard deviation (SD). No sex differences were identified in total bile acids, composition, or conjugation rate in any age group. *^1^ Bile acids in each group are listed in Supplementary Table S1. *^2^ Polyhydroxylated bile acids: CA-1β-ol, CA-6α-ol, and CDCA-1β-ol. *^3^ Other bile acids: 4-cholenoic, 4,6-cholandienoic, and 5-cholenoic acid derivatives (see Supplementary Table S1). *^4^ Unit, mmol/mol Cre; SD, standard deviation; m, months; y, years.

## Data Availability

The data presented in this study are available in the main article.

## Supplementary Materials

The following supporting information can be downloaded at: https://www.mdpi.com/article/10.3390/metabo12121230/s1, Table S1: Abbreviations and corresponding trivial names of glucuronidated, sulfated, unconjugated, metabolic intermediated, and polyhydroxylated bile acids considered in this study.

## References

[1] Bhatt D.K., Mehrotra A., Gaedigk A., Chapa R., Basit A., Zhang H., Choudhari P., Boberg M., Pearce R.E., Gaedigk R. (2019). Age- and Genotype-Dependent Variability in the Protein Abundance and Activity of Six Major Uridine Diphosphate-Glucuronosyltransferases in Human Liver. Clin. Pharmacol. Ther..

[2] Strassburg C.P., Strassburg A., Kneip S., Barut A., Tukey R.H., Rodeck B., Manns M.P. (2002). Developmental aspects of human hepatic drug glucuronidation in young children and adults. Gut.

[3] Neuvonen M., Hirvensalo P., Tornio A., Rago B., West M., Lazzaro S., Mathialagan S., Varma M., Cerny M.A., Costales C. (2020). Identification of Glycochenodeoxycholate 3-O-Glucuronide and Glycodeoxycholate 3-O-Glucuronide as Highly Sensitive and Specific OATP1B1 Biomarkers. Clin. Pharmacol. Ther..

[4] Sato K., Kakiyama G., Suzuki M., Naritaka N., Takei H., Sato H., Kimura A., Murai T., Kurosawa T., Pandak W.M. (2020). Changes in conjugated urinary bile acids across age groups. Steroids.

[5] Trottier J., Perreault M., Rudkowska I., Levy C., Dallaire-Theroux A., Verreault M., Caron P., Staels B., Vohl M.-C., Straka R.J. (2013). Profiling serum bile acid glucuronides in humans: Gender divergences, genetic determinants, and response to fenofibrate. Clin. Pharmacol. Ther..

[6] Takikawa H., Otsuka H., Beppu T., Seyama Y., Yamakawa T. (1982). Quantitative Determination of Bile Acid Glucuronides in Serum by Mass Fragmentography1. J. Biochem..

[7] Thakare R., Alamoudi J.A., Gautam N., Rodrigues A.D., Alnouti Y. (2018). Species differences in bile acids I. Plasma and urine bile acid composition. J. Appl. Toxicol..

[8] Ikegawa S., Okuyama H., Oohashi J., Murao N., Goto J. (1999). Separation and Detection of Bile Acid 24-Glucuronides in Human Urine by Liquid Chromatography Combined with Electrospray Ionization Mass Spectrometry. Anal. Sci..

[9] Perreault M., Wunsch E., Białek A., Trottier J., Verreault M., Caron P., Poirier G.G., Milkiewicz P., Barbier O. (2018). Urinary Elimination of Bile Acid Glucuronides under Severe Cholestatic Situations: Contribution of Hepatic and Renal Glucuronidation Reactions. Can. J. Gastroenterol. Hepatol..

[10] Knights K.M., Rowland A., Miners J.O. (2013). Renal drug metabolism in humans: The potential for drug–endobiotic interactions involving cytochrome P450 (CYP) and UDP-glucuronosyltransferase (UGT). Br. J. Clin. Pharmacol..

[11] Kimura A., Kagawa T., Takei H., Maruo Y., Sakugawa H., Sasaki T., Murai T., Naritaka N., Takikawa H., Nittono H. (2020). Rotor Syndrome: Glucuronidated Bile Acidemia From Defective Reuptake by Hepatocytes. Hepatol. Commun..

