# Supplementary material for: Profiling of Urinary Glucuronidated Bile Acids across Age Groups
Source: Metabolites. 2022 Dec 7;12(12):1230. doi: 10.3390/metabo12121230 (PMC9780789; doi:10.3390/metabo12121230)
Supplement: Supplementary file 1 [file metabolites-12-01230-s001.zip › metabolites-2064907-supplementary.pdf]

Supplemental Table S1: Abbreviations and corresponding trivial names of glucuronidated, sulfated, unconjugated, metabolic intermediated, and polyhydroxylated bile acids considered in this study

Abbreviations	Trivial names
**3-Glucuronidated, 24-Nonamidated**	
CA-3G	cholic acid 3-glucuronide
CDCA-3G	chenodeoxycholic acid 3-glucuronide
UDCA-3G	ursodeoxycholic acid 3-glucuronide
DCA-3G	deoxycholic acid 3-glucuronide
LCA-3G	lithocholic acid 3-glucuronide
HCA-3G	hyocholic acid 3-glucuronide
HDCA-3G	hyodeoxycholic acid 3-glucuronide
**3-Glucuronidated, 24-Glycine**	
GCA-3G	glycocholic acid 3-glucuronide
GCDCA-3G	glycochenodeoxycholic acid 3-glucucronide
GUDCA-3G	glycoursodeoxycholic acid 3-glucuronide
GDCA-3G	glycodeoxycholic acid 3-glucuronide
GLCA-3G	glycolithocholic acid 3-glucuronide
**3-Glucuronidated, 24-Taurine**	
TCA-3G	taurocholic acid 3-glucuronide
TCDCA-3G	taurochenodeoxycholic acid 3-glucuronide
TUDCA-3G	tauroursodeoxycholic acid 3-glucuronide
TDCA-3G	taurodeoxycholic acid 3-glucuronide
TLCA-3G	taurolithocholic acid 3-glucuronide
**3-Sulfated, 24-Nonamidated**	
CA-3S	cholic acid 3-sulfate
CDCA-3S	chenodeoxycholic acid 3-sulfate
UDCA-3S	ursodeoxycholic acid 3-sulfate
DCA-3S	deoxycholic acid 3-sulfate
LCA-3S	lithocholic acid 3-sulfate

**3-Sulfated, 24-Glycine**	
GCA-3S	glycocholic acid 3-sulfate
GCDCA-3S	glycochenodeoxycholic acid 3-sulfate
GUDCA-3S	glycoursodeoxycholic acid 3-sulfate
GDCA-3S	glycodeoxycholic acid 3-sulfate
GLCA-3S	glycolithocholic acid 3-sulfate
**3-Sulfated, 24-Taurine**	
TCA-3S	taurocholic acid 3-sulfate
TUDCA-3S	tauroursodeoxycholic acid 3-sulfate
TCDCA-3S	taurochenodeoxycholic acid 3-sulfate
TDCA-3S	taurodeoxycholic acid 3-sulfate
TLCA-3S	taurolithocholic acid 3-sulfate
**3-Unconjugated, 24-Nonamidated**	
CA	cholic acid
CDCA	chenodeoxycholic acid
UDCA	ursodeoxycholic acid
DCA	deoxycholic acid
LCA	lithocholic acid
HCA	hyocholic acid
**3-Unconjugated, 24-Glycine**	
GCA	glycocholic acid
GCDCA	glycochenodeoxycholic acid
GUDCA	glycoursodeoxycholic acid
GDCA	glycodeoxycholic acid
GLCA	glycolithocholic acid
GHCA	glycohyocholic acid
**3-Unconjugated, 24-Taurine**	
TCA	taurocholic acid
TCDCA	taurochenodeoxycholic acid
TUDCA	tauroursodeoxycholic acid
TDCA	taurodeoxycholic acid

TLCA	taurolithocholic acid
THCA	taurohyocholic acid
**Polyhydroxylated form**	
CA-1β-ol	1β,3α,7α,12α-tetrahydroxy-5β-cholanoic acid<br>(1β-hydroxy cholic acid)
GCA-1β-ol	glyco 1β,3α,7α,12α-tetrahydroxy-5β-cholanoic acid<br>(glyco1β-hydroxy cholic acid)
TCA-1β-ol	tauro 1β,3α,7α,12α-tetrahydroxy-5β-cholanoic acid<br>(tauro1β-hydroxy cholic acid)
CDCA-1β-ol	1β,3α,7α-trihydroxy-5β-cholanoic acid<br>(1β-hydroxy chenodeoxycholic acid)
CA-6α-ol	3α,6α,7α,12α-tetrahydroxy-5β-cholanoic acid<br>(6α-hydroxy cholic acid)
TCA-6α-ol	tauro 3α,6α,7α,12α-tetrahydroxy-5β-cholanoic acid<br>(tauro 6α-hydroxy cholic acid)
GCA-6α-ol	glyco 3α,6α,7α,12α-tetrahydroxy-5β-cholanoic acid<br>(glyco 6α-hydroxy cholic acid)
**Others (Metabolic intermediate related)**	
CA-Δ^{4}-3-one	7α,12α-dihydroxy-3-oxo-4-cholenoic acid
GCA-Δ^{4}-3-one	glyco 7α,12α-dihydroxy-3-oxo-4-cholenoic acid
TCA-Δ^{4}-3-one	tauro 7α,12α-dihydroxy-3-oxo-4-cholenoic acid
CDCA-Δ^{4}-3-one	7α-hydroxy-3-oxo-4-cholenoic acid
GCDCA-Δ^{4}-3-one	glyco 7α-hydroxy-3-oxo-4-cholenoic acid
TCDCA-Δ^{4}-3-one	tauro 7α-hydroxy-3-oxo-4-cholenoic acid
DCA-$\Delta^{4,6}$-3-one	12α-hydroxy-3-oxo-4,6-choladienoic acid
LCA-$\Delta^{4,6}$-3-one	3-oxo-4,6-choladienoic acid
Δ^{5}-3β-ol	3β-hydroxy-5-cholenoic acid
GΔ^{5}-3β-ol	glyco 3β-hydroxy-5-cholenoic acid
TΔ^{5}-3β-ol	tauro 3β-hydroxy-5-cholenoic acid
Δ^{5}-3β-ol-3S	3β-hydroxy-5-cholenoic acid 3-sulfate
GΔ^{5}-3β-ol-3S	glyco 3β-hydroxy-5-cholenoic acid 3-sulfate
TΔ^{5}-3β-ol-3S	tauro 3β-hydroxy-5-cholenooic acid 3-sulfate
Δ^{5}-3β,7α-diol	3β,7α-dihydroxy-5-cholenoic acid

GΔ^5-3β,7α-diol	glyco 3β,7α-dihydroxy-5-cholenoic acid
TΔ^5-3β,7α-diol	tauro 3β,7α-dihydroxy-5-cholenoic acid
Δ^5-3β,7α-diol-3S	3β,7α-dihydroxy-5-cholen-24-oic acid 3-sulfate
GΔ^5-3β,7α-diol-3S	glyco 3β,7α-dihydroxy-5-cholenoic acid 3-sulfate
TΔ^5-3β,7α-diol-3S	tauro 3β,7α-dihydroxy-5-cholenoic acid 3-sulfate
Δ^5-3β,7α,12α-triol	3β,7α,12α-trihydroxy-5-cholenoic acid
GΔ^5-3β,7α12α-triol	glyco 3β,7α,12α-trihydroxy-5-cholenoic acid
TΔ^5-3β,7α12α-triol	tauro 3β,7α,12α-trihydroxy-5-cholenoic acid
Δ^5-3β,7α,12α-triol-3S	3β,7α,12α-trihydroxy-5-cholen-24-oic acid 3-sulfate
GΔ^5-3β,7α12α-triol-3S	glyco 3β,7α,12α-trihydroxy-5-cholenoic acid 3-sulfate
TΔ^5-3β,7α12α-triol-3S	tauro 3β,7α,12α-trihydroxy-5-cholenoic acid 3-sulfate
G-nor-CA	glyconorcholic acid
T-nor-CA	tauronorcholic acid
nor-CA	norcholic acid
C_{27}-DHCA	3α,7α-dihydroxycholestanoic acid
C_{27}-THCA	3α,7α,12α-trihydroxycholestanoic acid
GΔ^5-3β,7β- diol-3S-7NAG	glyco 3β,7β-dihydroxy-5-cholenoic acid 3-sulfate 7-N-acetylglu-cosaminide
TΔ^5-3β,7β- diol-3S-7NAG	tauro 3β,7β-dihydroxy-5-cholenoic acid 3-sulfate 7-N-acetylglu-cosaminide
Δ^5-3β,7β- diol-3S-7NAG	3β,7β-dihydroxy-5-cholenoic acid 3-sulfate 7-N-acetylglu-cosaminide
Δ^5-3β,7β-diol-3S	3β,7β-dihydroxy-5-cholenoic acid 3-sulfate
